# Non-Invasive Measurement Using Deep Learning Algorithm Based on Multi-Source Features Fusion to Predict PD-L1 Expression and Survival in NSCLC

**DOI:** 10.3389/fimmu.2022.828560

**Published:** 2022-04-07

**Authors:** Chengdi Wang, Jiechao Ma, Jun Shao, Shu Zhang, Jingwei Li, Junpeng Yan, Zhehao Zhao, Congchen Bai, Yizhou Yu, Weimin Li

**Affiliations:** ^1^ Department of Respiratory and Critical Care Medicine, Med-X Center for Manufacturing, Frontiers Science Center for Disease-Related Molecular Network, West China Hospital, West China School of Medicine, Sichuan University, Chengdu, China; ^2^ AI Lab, Deepwise Healthcare, Beijing, China; ^3^ West China Hospital, West China School of Medicine, Sichuan University, Chengdu, China; ^4^ Department of Medical Informatics, West China Hospital, Sichuan University, Chengdu, China; ^5^ Department of Computer Science, The University of Hong Kong, Pokfulam, Hong Kong SAR, China

**Keywords:** deep learning, PD-L1 expression, survival, lung cancer, radiomics

## Abstract

**Background:**

Programmed death-ligand 1 (PD-L1) assessment of lung cancer in immunohistochemical assays was only approved diagnostic biomarker for immunotherapy. But the tumor proportion score (TPS) of PD-L1 was challenging owing to invasive sampling and intertumoral heterogeneity. There was a strong demand for the development of an artificial intelligence (AI) system to measure PD-L1 expression signature (ES) non-invasively.

**Methods:**

We developed an AI system using deep learning (DL), radiomics and combination models based on computed tomography (CT) images of 1,135 non-small cell lung cancer (NSCLC) patients with PD-L1 status. The deep learning feature was obtained through a 3D ResNet as the feature map extractor and the specialized classifier was constructed for the prediction and evaluation tasks. Then, a Cox proportional-hazards model combined with clinical factors and PD-L1 ES was utilized to evaluate prognosis in survival cohort.

**Results:**

The combination model achieved a robust high-performance with area under the receiver operating characteristic curves (AUCs) of 0.950 (95% CI, 0.938–0.960), 0.934 (95% CI, 0.906–0.964), and 0.946 (95% CI, 0.933–0.958), for predicting PD-L1ES <1%, 1–49%, and ≥50% in validation cohort, respectively. Additionally, when combination model was trained on multi-source features the performance of overall survival evaluation (C-index: 0.89) could be superior compared to these of the clinical model alone (C-index: 0.86).

**Conclusion:**

A non-invasive measurement using deep learning was proposed to access PD-L1 expression and survival outcomes of NSCLC. This study also indicated that deep learning model combined with clinical characteristics improved prediction capabilities, which would assist physicians in making rapid decision on clinical treatment options.

## Introduction

Lung cancer, one of the most common types of cancer, occupies the leading cause of malignant mortality (1). In China, only 17.3% of lung cancer patients were stage I at primary diagnosis and 5-year survival was under 20% (2). On the basis of its histological types, non-small cell lung cancer (NSCLC) accounts for about 85% of all cases (3). It is a critical trend to develop individualized therapeutic schemes based on comprehensive usage of multiple-modality data for NSCLC patients.

Programmed cell death-ligand 1 (PD-L1), a ligand of programmed cell death 1 (PD-1), frequently overexpressed on the surface of cancer cells to invade the anti-tumor immunity through PD-1/PD-L1 pathway (4). Thus, numerous clinical trials were conducted to evaluate the efficacy of immunotherapy targeted PD-1/PD-L1 pathway (5–7). However, the IMpower150 trial indicated high expression (≥50%) of PD-L1 showed a better outcome for NSCLC received atezolizumab than lower expression of PD-L1 (6). Furthermore, KEYNOTE-042 presented the similar results (7). Thus, National Comprehensive Cancer Network (NCCN) Guidelines proposed that immunohistochemistry should be applied to assess the expression of PD-L1 and further guide immunotherapy (8). Nevertheless, immunohistochemistry requires invasive procedure to obtain the samples of NSCLC, and sample quality influences the results of PD-L1 expression to a great extent (9). Thus, other alternative methods to measure the PD-L1 status would greatly assist clinical decision support, especially for insufficient available samples or immunohistochemistry failure.

Recently, state-of-the-art artificial intelligence (AI) methods such as deep learning have been applied to screen lung cancer, assist the drug efficacy and prognosis prediction (10–13). Previous study used deep learning on whole-slide images to assist pathologists in the precise assessment of immunotherapy-related biomarkers (14). However, it still remains a challenging issue of invasive, intertumoral heterogeneity and dynamic changes. As a convenience and one that is routinely available in clinical practice, CT images reflect the whole information of an entire tumor non-invasively. A recent study has constructed deep learning model to predict PD-L1 expression (≥50%) with high performance (area under the receiver operating characteristic curve, AUC ≥0.71), which could help to predict the efficacy of immunotherapy in patients with NSCLC and indicate a direction for molecular prediction utilizing deep learning (15). However, previous studies mainly classified binary patterns on basis of relatively small samples, and the prediction of detailed classification were warranted for further investigation.

Thus, we proposed a non-invasive deep learning model based on radiomic signature from pretreatment CT images to predict the PD-L1 status and overall survival (OS) in lung cancer, guiding the clinical practice.

## Methods

### Patients’ Cohort and Data Collection

This retrospective study was approved by the institutional research board of the West China Hospital of Sichuan University. A total of 1,274 non-small lung cancer patients diagnosed between January, 2016, and April, 2019 were enrolled in this study. Written informed consent was waived because the data used for system development were anonymized by removing personal information. All patients met the following inclusion criteria: 1) pathological analysis of tumor tissues confirmed non-small cell lung cancer; 2) chest CT examinations were available; and 3) PD-L1 expression signature (PD-L1ES) was detected (<1%, 1–49%, ≥50%) in immunohistochemical (IHC) assays performed on the Ventana Benchmark platform (SP142 antibody). Patients were excluded if: 1) basic clinical information (such as age, sex, and PD-L1ES) were missing; 2) preoperative treatment or the time between CT examination and subsequent surgery exceeded 1 month; 3) CT images were of low quality; and 4) molecular testing results were difficult to determine. After screening with the exclusion criteria, the PD-L1ES prediction cohort (n = 1,135) for classifying PD-L1 expression signature was built (**Supplementary Figure 1**). Moreover, the survival cohort was created to examine the association between various factors and prognosis prediction. The inclusion criteria for building the survival cohort were: 1) clear clinical information was provided including smoking history, family history, histopathology such as lung adenocarcinoma (LUAD), squamous cell carcinoma (LUSC), etc.; 2) treatment status (surgery, chemotherapy, radiation therapy, targeted therapy, immunotherapy etc.) and survival outcome were all gathered from medical records or telephone follow-up. Finally, the survival cohort included 811 patients who were admitted to the West China Hospital of Sichuan University and followed up to June 2021.

### Data Pre-Processing

This study consisted of two datasets: the PD-L1ES prediction dataset (n = 1,135) and the survival dataset (n = 811). The PD-L1ES dataset was divided into three categories based on different levels of PD-L1ES, namely, low PD-L1ES <1%, medium PD-L1ES 1–49%, and high PD-L1ES ≥50%, aiming to predict molecular events based on PD-L1 expression status. The survival cohort was utilized to create an accessing model to investigate the association between survival risk factors and overall survival (OS). In this study, the training and testing cohorts from both datasets were randomly partitioned with a ratio of 4:1, and five-fold cross validations were used for model development and validation.

To address the imbalance of three PD-L1ES groups, we adopted a joint re-weight strategy and re-sampling approach, such as up-sampling the short-tail instances and down-sampling the long-tail ones. Moreover, in order to enhance the learning capacities of the developed models, we employed a combination of tactics (radiomics model: low-level feature; deep learning model: high-level feature, clinical model: semantic feature). Our combination model extracted CT-based deep learning features, CT-based radiomic features, and clinical record-based clinical data for each patient. Two groups of experienced radiologist specialists delineated the particular contour of the entire tumor and the region of interest (ROI) for that tumor was determined accordingly. All ROIs were resized to the same resolution of 36 ∗ 36 ∗ 36 cubes for deep feature extraction using third-order spline interpolation and normalized to 0–255 using pre-computed windowing information of lung. To make the full use of the enrolled data for clinical information extraction, the k-nearest neighbor (KNN) technique was first utilized to impute missing clinical values.

### Establishment of the Low-Level Feature Model

For each tumor cube and its paired segmentation mask, a dedicated open-source Python library called PyRadiomics (http://www.radiomics.io; v3.0.1) was applied to perform radiomic phenotypic feature extraction from the three-dimensional ROI. Our study used it to automatically extract 1,672 radiomic characteristics for each cube-mask pair, namely, first-order, size, shape, and texture features. The least absolute shrinkage and selection operator (LASSO) method was applied for feature dimensionality reduction to select radiomic features with high stability and repeatability of radiological properties. L1 penalty was used to shrink some regression coefficients to exactly zero. Following that, we calculated the partial probability deviation on each radiomic characteristics of that tumor, and removed radiomic characteristics with low variance (less than 0.6), yielding a total of 107 features for each cube-mask pair, comprising of first-order (18 features), shape (14 features), and texture (75 features).

We established a gradient boosting regressor on the extracted radiomic features for survival analysis. Gradient boosting was a traditional machine-learning approach used in high-dimensional data analysis for classification and regression applications. It was an ensemble model that comprised of numerous weak learners and output the sum of their results. Based on correcting the error of the predecessor learner, each weak prediction model approximated a feature screening function. The gradient descent approach gave guidance for the direction of the ensemble model’s development, allowing the model to attain higher and more resilient performance. We utilized the coefficient of determination (R2) score as an assessment metric to determine the parameters of the model on our dataset. A higher R2 score (from 0 to 1) indicated greater consistency between the predictive value with the ground truth. Through a series of tests, hyper-parameter optimization was carried out in line with the assessment outcomes of both training and test set data. As a result, the generalization capability of the regressor could be ensured, and we finally acquired the radiomic features.

To comprehensively investigate the performance of several classifiers in the PD-L1ES prediction challenge, we also combined the aforementioned radiomic feature with other imaging features to predict the PD-L1ES. While the random survival forest machine-learning technique was used to generate the radiomics model and a radiomics score (Rad-score) for survival analysis and risk stratification. A higher Rad-score indicated a higher risk of progression or a shorter overall survival, representing the average projected number of events across all trees in the constructed forest.

### Establishment of the High-Level Feature Model

In our study, the high-level feature model also had two components: a 3D ResNet model as the feature extractor to obtain deep learning features and the specialized classifier/regressor for the prediction task.

The deep learning feature extraction module received the ROI cubes of the CT image as input. Compared to general 2D image processing, 3D ResNet could take 3D context into consideration and capture more complementary information from various slices of the same tumor, and thus make better decision given the 3D features. Pertaining to the small-sized 3D tumor volume, we modified the traditional 3D ResNet by neglecting the sub-sample operation in the conv stem layers so as to retain as much detailed texture information as possible. Finally, ResNet totally sub-sample to 1/8 of the original input compared to 1/32 in the vanilla ResNet. Transfer learning was also applied in our deep feature extraction model, where model parameters learned from large scale data from another domain were used to aid our target learning tasks (prediction and evaluation tasks) in a new environment. Such an approach would minimize the number of training epochs, expedite model convergence, and, to some extent, prevent over-fitting. The deep learning features obtained from the PD-L1ES prediction task were also employed for the evaluation task afterwards.

We built a 3D ResNet to predict different levels of PD-L1 expression signature (low PD-L1ES, medium PD-L1ES, and high PD-L1ES). The 3D ResNet was composed of a feature extraction backbone and a classification head. The classification head used a binary cross-entropy loss function to back-propagate the loss error. All the layers in the 3D ResNet backbone were initialized with pre-trained weights, while other parameters were randomly initialized. We trained all models for 40 epochs. The learning rate was set to 0.02 and decayed by a factor of 10 after the 20th and 35th epochs respectively.

In addition, we used the trained 3D ResNet to extract deep learning features to retrieve the survival risk and integrated it with other mined features to create the combination model for the prognosis task. From these stable features, the deep learning features that contributed the most to OS were chosen to provide a deep learning score (DL-score), and an ideal score was cut-off using the X-tile software to distinguish between high and low progression risk.

### Establishment of the Multi-Source Features Fusion Model

**Figure 1:**
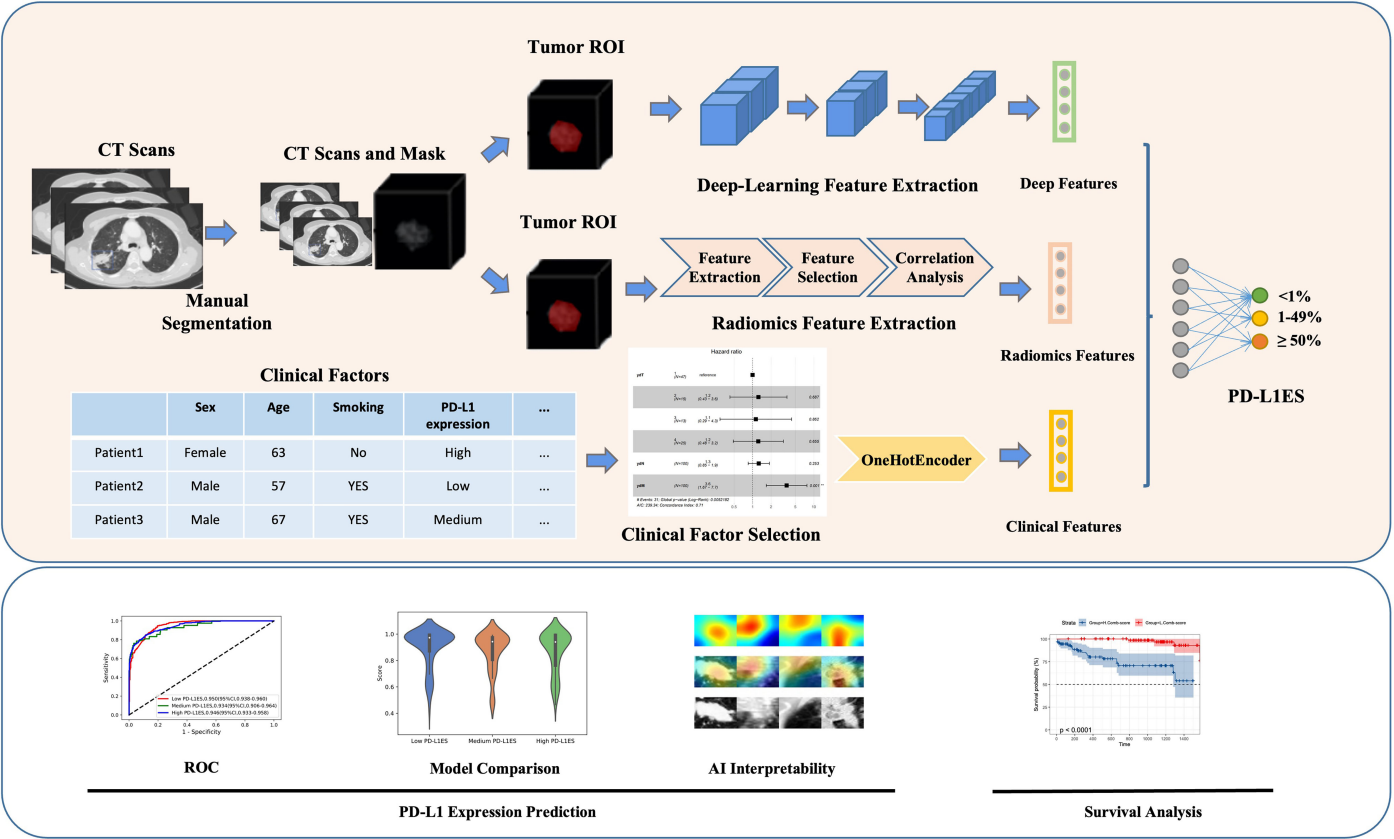
Overall workflow of study design. The upper part showed the overall method of the study, while the lower part represented the analysis of the model. Original CT imaging data with manually labeled tumor images, comprehensive patient clinical information, overall survival, and PD-L1 expression signature were included in the data processing step. The deep learning, radiomics and clinical features were retrieved using the tumor ROI or clinical records during the feature extraction step. All features were utilized to predict PD-L1 expression signature and evaluate patient survival. PD-L1ES, PD-L1 expression signature; ROI, region of interest.

In this study, two tasks were performed: PD-L1ES prediction and the prognosis evaluation (**Figure 1**). Besides the extracted imaging features, the clinical features were employed to offer extra semantic information for both tasks. To identify potential important variables in the clinical feature, a Kaplan–Meier curve with log-rank test and a univariate Cox proportional-hazards model were used (clinical factors and PD-L1ES). Factors with a *P*-value <0.05 were included to format the clinical features. Factors were transferred to one-hot encoding into a high-dimensional vector space for the prediction task, and they were employed in the multivariate Cox proportional-hazards regression to build the clinical model for the survival analysis task.

We totally established two combination model (multi-source features model), one for the PD-L1ES identification task and the other for the survival analysis task. For the PD-L1ES prediction task based on the deep learning features, radiomic features and the one-hot clinical features were fused by a self-adapted fully-connected layer to generate the fusion embedding. For the prognosis prediction task, the deep learning features and the radiomic features were treated as Rad-score and DL-score to build the univariate for risk stratification. Then, to build the combination model, a multivariate Cox proportional-hazards model with the lowest Akaike information criteria was used to combine the Rad-score, DL-score, and high-relevance clinical risk variables.

### Statistical Analysis

The following measures were used to assess the performance of classifiers: AUC, accuracy, sensitivity, and specificity. The DeLong technique was used to calculate the 95% confidence intervals (CIs) for the AUC. All statistical tests were two-tailed, with statistical significance set at *P <*0.05 was considered as significant. OS, measured from the diagnosis to death or the last follow-up, was generated using the Kaplan–Meier method, and differences in OS were examined using the log-rank test. Hazard ratios (HRs) were presented with their 95% CIs. The risk stratification capability was assessed by using the Kaplan–Meier survival curve and log-rank test. The optimal Rad-score and DL-score cut-off values were determined by X-tile software (version 3.6.1, Yale University).

## Results

### Clinical Characteristics

After screening 1,274 patients with lung cancer who had molecular test, a total of 1,135 eligible patients who met the criteria were identified in prediction cohort, namely, 722 (63.6%) patients with PD-L1ES <1%, 50 (4.4%) patients with PD-L1ES 1–49%, and 363 (32.0%) patients with PD-L1ES ≥50% (**Supplementary Figure 1**). The mean age was 58.77 ± 10.66 years (**Table 1**). Women were predominant (582/1135, 51.3%), and more than 60% were never smokers. In terms of treatment, 784 patients (69.1%) received surgery, while 222 patients (19.6%) received radiation. Furthermore, 295 patients received chemotherapy and 386 patients receiving targeted treatment accounted for 26% and 34%, respectively. In terms of TNM stage, the majority of patients were in stage I (n = 498, 43.9%) and stage IV (n = 321, 28.3%), respectively. According to the data split strategy, 908 patients were utilized for training and internal validation, and 227 patients were used for external validation. Meanwhile, PD-L1ES survival cohort included 811 patients with complete follow-up information (**Supplementary Figure 1** and **Table 1**).

**Table 1:** Clinical characteristics of patients used to measure PD-L1ES and survival analysis.

	Whole Cohort (n = 1,135)	Survival Cohort (n = 811)
**Age (years)**	58.77 ± 10.66	57.80 ± 11.03
**Sex, N (%)**		
Male	553 (48.7%)	371 (45.7%)
Female	582 (51.3%)	440 (54.3%)
**Smoking status**		
Current or former	403 (35.5%)	265 (32.7%)
Never	692 (61.0%)	517 (63.7%)
Unknown	40 (3.5%)	29 (3.6%)
**Family history of cancer**		
Yes	92 (8.1%)	94 (11.6%)
No	1,026 (90.4%)	715 (88.2%)
Unknown	17 (1.5%)	2 (0.2%)
**Surgery**		
Yes	784 (69.1%)	558 (68.8%)
No	351 (30.9%)	253 (31.2%)
**Radiotherapy**		
Yes	222 (19.6%)	161 (19.8%)
No	913 (80.4%)	650 (80.2%)
**Chemotherapy**		
Yes	295 (26.0%)	318 (39.2%)
No	508 (44.8%)	482 (59.4%)
Unknown	332 (29.2%)	11 (1.4%)
**Targeted therapy**		
Yes	386 (34.0%)	296 (36.5%)
No	749 (66.0%)	515 (63.5%)
**Immunotherapy**		
Yes	30 (2.6%)	27 (3.3%)
No	1,105 (97.4%)	784 (96.7%)
**Histopathology**		
LUAD	1,038 (91.4%)	755 (93.1%)
LUSC	36 (3.2%)	31 (3.8%)
Other	61 (5.4%)	25 (3.1%)
**Stage T**		
Tis	2 (0.2%)	3 (0.4%)
T1	440 (38.8%)	323 (39.8%)
T2	368 (32.4%)	258 (31.8%)
T3	100 (8.8%)	74 (9.1%)
T4	171 (15.1%)	118 (14.6%)
Tx	54 (4.7%)	35 (4.3%)
**Stage N**		
N0	603 (53.1%)	444 (54.7%)
N1	83 (7.3%)	59 (7.3%)
N2	240 (21.1%)	164 (20.2%)
N3	121 (10.7%)	84 (10.4%)
Nx	88 (7.8%)	60(7.4%)
**Stage M**		
M0	781 (68.8%)	559 (68.9%)
M1	300 (26.4%)	219 (27.0%)
Mx	54 (4.8%)	33 (4.1%)
**Stage**		
I	498 (43.9%)	363 (44.8%)
II	95 (8.3%)	63 (7.8%)
III	204 (18.0%)	142 (17.5%)
IV	321 (28.3%)	234 (28.8%)
Unknown	17 (1.5%)	9 (1.1%)
**PD-L1 ES (%)**		
<1%	722 (63.6%)	481 (59.3%)
1–49%	50 (4.4%)	49 (6.0%)
≥50%	363 (32.0%)	281 (34.7%)

LUAD, lung adenocarcinoma; LUSC, lung squamous cell carcinoma; ES, expression signature.

### Detection of PD-L1 Expression Status

**Figure 2:**
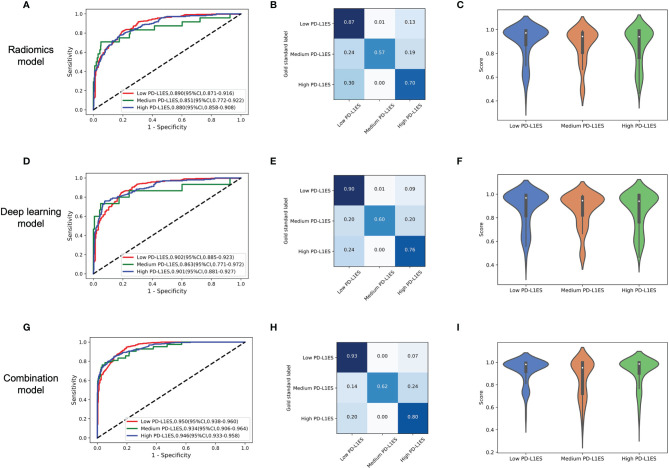
The model performance in the prediction of PD-L1ES. The figure contained three groups of result analysis, including ROC curve, confusion matrix, and score distribution. The red line depicted low PD-L1 expression performance, the green line depicted medium PD-L1 expression performance, and the blue line depicted high PD-L1 expression performance. **(A–C)** The performance of the radiomics model. **(D–F)** The performance of the deep learning model. **(G–I)** The performance of the combination model. PD-L1ES, PD-L1 expression signature.

The combination model achieved excellent performance to predict PD-L1 expression status than radiomics and deep learning methods (**Figure 2**). The ROC curve, confusion matrix and the model score distribution of PD-L1ES classification illustrated the performance of radiomics model with AUCs of low PD-L1ES 0.890 (95% CI, 0.871–0.916), medium PD-L1ES 0.851 (95% CI, 0.772–0.922), and high PD-L1ES 0.880 (95% CI, 0.858–0.908), respectively. Meanwhile, the deep learning model yielded AUCs of 0.902 (95% CI, 0.885–0.923), 0.863 (95% CI, 0.771–0.972), and 0.901 (95% CI, 0.881–0.927) for PD-L1ES <1%, 1–49%, and ≥50% in validation cohort, respectively. The AUC of the combination model also have the appreciable effect of low PD-L1ES 0.950 (95% CI, 0.938–0.960), medium PD-L1ES 0.934 (95% CI, 0.906–0.964), and high PD-L1ES 0.946 (95% CI, 0.933–0.958). The violin diagram of model score indicated that the predictive performance of deep learning was better than the performance of the radiomics model, and the combination model achieved the best predictive performance in terms of the PD-L1 ES identification.

**Figure 3:**
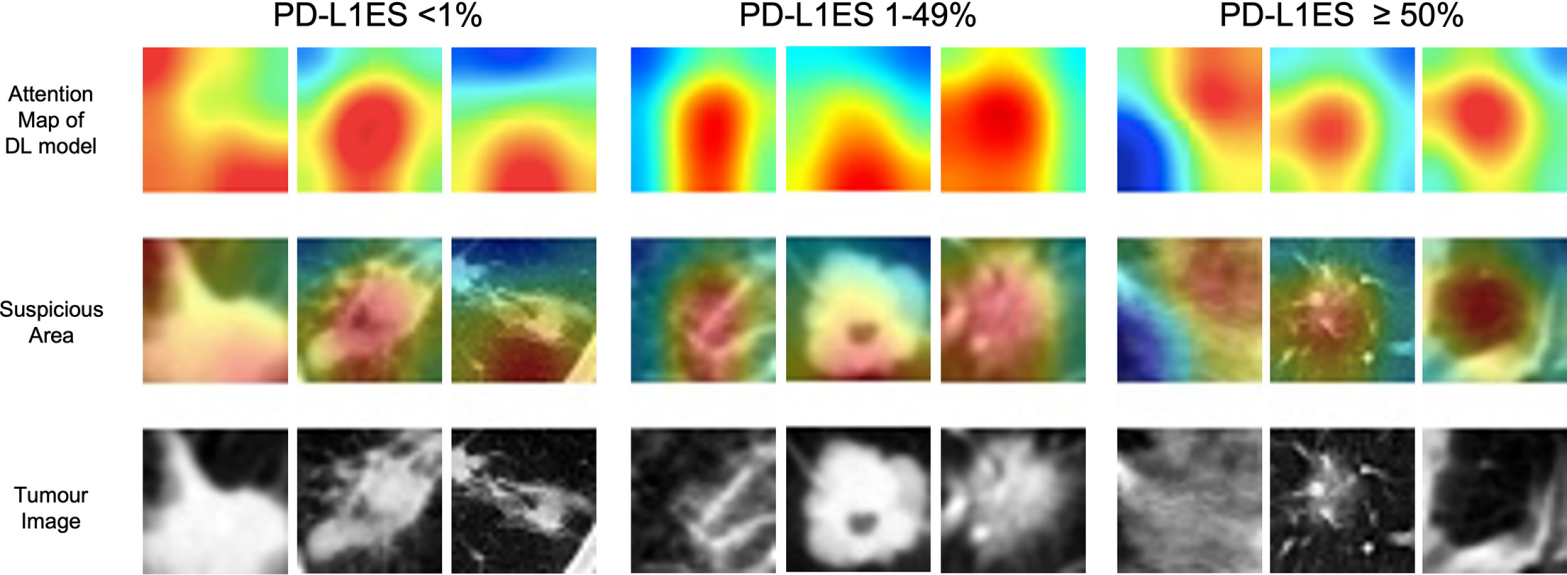
The illustration of deep learning feature heatmap to predict PD-L1 expression. The first and second rows visualize the attention regions of a network for distinct mutant categories; the third row shows the origin tumor image in the 3D volume. PD-L1ES, PD-L1 expression signature.

Through the CAM visualization of the prediction model, the center of tumor region was identified as an important area for PD-L1 status classification with darker response color (**Figure 3**). It may provide clinicians with a biopsy position to as much as possible avoid misdiagnosis caused by intra-tumor heterogeneity.

### Exploring Characteristics Associated With OS

**Figure 4:**
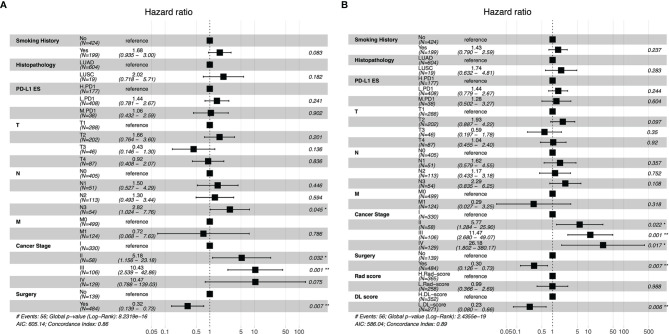
Forest plots of clinical model **(A)**, the combination model with radiomics score and deep learning score **(B)**. PD-L1ES, PD-L1 expression signature; LUAD, lung adenocarcinoma; LUSC, lung squamous cell carcinoma; Rad, radiomics; DL, deep learning.

According to the Kaplan–Meier survival analysis (**Supplementary Figure 2**), smoking history (*P <*0.001), surgery (*P <*0.001), chemotherapy (*P <*0.001), TMN stage (*P <*0.001), and cancer stage (*P <*0.001) were all significantly associated with OS. Patients with a smoking history had a considerably lower OS than those with no smoking history, and patients who had surgery had a significantly superior OS than those who did not. Then, taking into account the influence of clinical factors on OS, we built the clinical model using forest plots (**Figure 4A**) and selected the hazard ratio correlation with patient survival, followed by the multivariate analysis cox proportional-hazards regression (C-index of clinical model: 0.86). The clinical model was then verified using subgroup stratified analysis to see whether it could predict prognosis in individuals with various PD-L1ES. The patients in each group were classified by regression score as high or low risk of disease progression.

### Validation of the PD-L1ES in Survival Analysis

**Figure 5:**
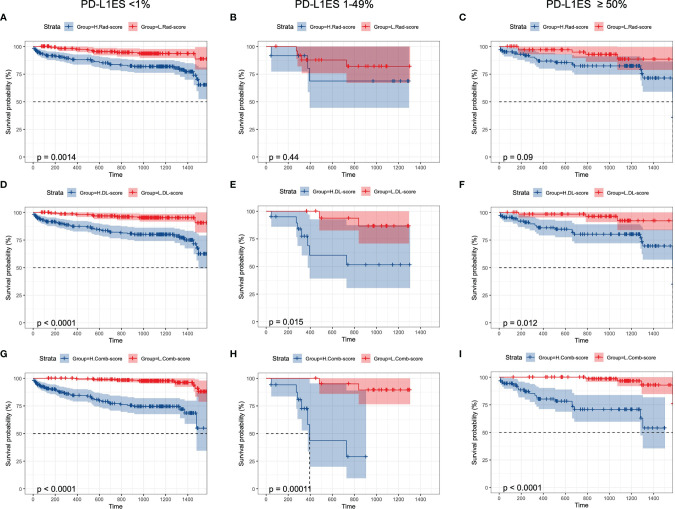
Kaplan–Meier curves of overall survival prediction in different PD-L1 ES groups. K–M curves were stratified by **(A–C)** Rad-score, **(D–F)** DL-score, **(G–I)**: combination-score to identify high-risk and low-risk groups. PD-L1ES, PD-L1 expression signature; Rad, radiomics; DL, deep learning.

Rad-score, DL-score, and clinical relevant features were used to create the combination model. We employed the multivariate Cox regression model to assess the importance of these characteristics in predicting OS. Furthermore, the forest plots of the combination model showed that cancer stage (HR = 26.18, *P* = 0.017), surgery (HR = 0.30, *P* = 0.007) and DL-score (HR = 0.23, *P* = 0.006) were independent predictors for OS. The performance of the combination model with a C-index of 0.89 was superior than the clinical model with a C-index of 0.86 (**Figures 4A, B**). Prognostic performance in different subgroups was compared by different models of low PD-L1ES <1%, medium PD-L1ES 1–49% and high PD-L1ES ≥50% to stratify the subgroup into low-risk and high-risk groups (**Figure 5**). The deep learning model score and combination model score achieved excellent discriminative ability regardless of three groups (all *P <*0.05).

## Discussion

According to the NCCN guidelines, PD-L1 expression levels of patients with lung cancer have a marked effect on clinical decision whether to use single-agent immunotherapy or interpretation chemotherapy (8). In this study, we constructed an alternative non-invasive measurement of accessing PD-L1 status and a prognostic model of predicting OS with high accuracy. It would be of paramount importance on clinical decision support, especially when tissues were not available.

The paradigm treatment of lung cancer patients varied according to different status of PD-L1 (16). The level of PD-L1 expression was presented as TPS given as <1%, 1–49%, and ≥50%. Atezolizumab single treatment was the preferred recommendation for patients with a PD-L1 expression status of ≥50% (17). While nivolumab combined with ipilimumab was first-line treatment for patients with PD-L1 expression status of ≥1% (18, 19). At the same time, PD-L1 expression status were useful for deciding whether to use single-agent immunotherapy or immunotherapy combinations as well (20, 21). Therefore, the study defined 1% and 50% as cut-off values. The predictive model could accurately evaluate the expression of three classifications, which was more in line with the clinical standard treatment needs.

Conventional assessment of PD-L1 TPS was a time-consuming task due to intertumoral heterogeneity, inter-observer variability, and different antibodies with various possible staining properties (22, 23). As a non-invasive and repeatable method, deep learning based on CT images provided a novel solution. Previous studies have confirmed the application of radiomics and deep learning on accessing PD-L1 status (**Table 2**) (15, 24–31). The latest research proposed a small-residual-convolutional-network (SResCNN) of PET/CT images to discriminate between PD-L1 positive and negative patients (1% cut-off, Dako22C3 antibody) with AUC of ≥0.82 in the training, validation, and testing cohorts (24). The current work was the single largest study population of patients with NSCLC and achieved excellent performance of prediction. However, the challenges of AI faced on the path to clinical adoption cannot be ignored. For instance, the interpretability hindered the widespread of the models. In this study, we utilized heatmap for visualization of deep learning, but the principle of pathophysiology was still inexplicable. Several studies indicated high-response areas of deep learning model based on PET/CT images to predict PD-L1 ES could recognize the necrotic region of lung cancer (24, 32). One possible explanation was that hypoxia leaded cell necrosis while upregulating PD-L1 through hypoxia-inducible factor 1α (33). Therefore, continuous optimization and exploration were warranted for AI-based software to improve patient care ultimately.

**Table 2:** Recent studies of predicting PD-L1 status on CT images by radiomic or deep learning in lung cancer patients.

First author, Year	Model	Imaging modality	PD-L1 assays	Cut-off	Population,	Performance
Wei Mu (24)	DL small- residual-convolutional-network (SResCNN)	PET/CT	22C3	1%	485 NSCLCs to measure PD-L1 status, 284/116/85 for training validation/testing cohort	AUC of 0.89 (95% CI: 0.84 to 0.94), 0.84 (95% CI: 0.76 to 0.92);, and 0.82 in the training, validation, and testing cohorts, respectively
Panwen Tian (15)	DL KNN	CT	SP142	50%	939 NSCLCs, 750/93/96 for training validation/testing cohort	AUC of 0.78, 0.71, and 0.76 in the training, validation, and testing cohorts
Ying Zhu (25)	DL CNN 3D DenseNets	CT	SP263	1%, 50%	127 advanced LUADs, five-fold cross-validation	1%, AUC of 0.784; 50%, AUC of 0.765
Qiang Wen (26)	Radiomics	CT	SP263	50%	120 advanced NSCLCs	AUC of 0.730 based on radiomic signatures, AUC of 0.839 combined radiomic signatures with clinical and morphological factors
Zekun Jiang (27)	Radiomics	CT	NA	1%	125 NSCLC	AUC of 0.96, 0.85 in training, validation cohort
Stefano Bracci (28)	Radiomics	CT	SP263	50%	72 advanced NSCLCs	AUC of 0.811 and 0.789 in the training and validation cohort
Zongqiong Sun (29)	Radiomics	CT	22C3	50%	390 NSCLC, 260/130 for training/validation cohort	AUC of 0.829 and 0.848 in the training and validation cohort
Jiyoung Yoon (30)	Radiomics	CT	SP263	50%	153 advanced LUADs	AUC of 0.661 (95% CI 0.580–0.735)
Mengmeng Jiang (31)	Radiomics	CT, PET/CT	SP142	1%, 50%	399 stage I–IV NSCLCs	1%, AUC of 0.97, 0.61, and 0.97 in the CT, the PET, and the PET/CT images respectively; 50% AUC of 0.80, 0.65, and 0.77
			28-8			1%, AUC of 0.86, 0.62, and 0.85; 50%, AUCs of 0.91, 0.75, and 0.88

DL, deep learning; AUC, area under the curve; LUAD, lung adenocarcinoma; NSCLC, Non-small cell lung cancer; CNN, convolutional neural network; KNN, k-nearest neighbor; NA, not applicable.

Although PD-L1 expression level was widely used, it was not an ideal biomarker for treatment efficacy and prediction model. A phase III randomized trial suggested that TMB might be a useful immune biomarker for deciding whether to use immunotherapy in patients with metastatic NSCLC (18). Moreover, a radiomic signature for CD8 cells that included eight variable CD8 cell radiation signatures containing eight variables to infer clinical outcomes for patients with cancer who had been treated with anti-PD-1/PD-L1 (34). In this study, prognostic models based on usual clinical characteristics or deep learning features were established. Only the comprehensive model combined Rad-score, DL-score, and clinically relevant features achieved superior prediction performance. This indicated the complementarity of radiology image features and clinical variables, and potential of deep learning in precise prognostic assessment.

The current study has some limitations. First of all, this was a single-center study with patients tested with SP42. The model required more central data for verification. Second, this retrospective research cannot avoid the biases of patient selection and detection results. For example, the small number of PD-L1 ES 1-49% group may limit the performance of model. Unfortunately, due to the small number of immunotherapy treatments, we did not predict the efficacy of immune checkpoint inhibitors, which was the direction of our future efforts. Thirdly, this study focused on multi-class prediction for PD-L1 expression, which was unable to cover all actionable biomarkers. Large-scale prospective samples from multi-centers are warranted for investigation in the future.

## Conclusion

In conclusion, a non-invasive method to measure PD-L1 expression status and infer clinical outcomes was proposed. This deep learning model combined with clinical variables has showed excellent performance, which has potential to effectively manage the personalized treatment of NSCLC patients.

## Data Availability Statement

The original contributions presented in the study are included in the article/**Supplementary Material**. Further inquiries can be directed to the corresponding authors.

## Ethics Statement

The studies involving human participants were reviewed and approved by the Ethics approval was obtained from the ethics committee of West China Hospital. The patients/participants provided their written informed consent to participate in this study.

## Author Contributions

WML and YZY were involved in the study design. CDW, JCM, and JS were involved in the organization of the entire project, data analysis with a clinical perspective, and manuscript writing. CDW, JS, JWL, ZHZ and CCB collected the imaging and clinical data. JCM, JPY, and SZ were involved in the establishment of the algorithm. All authors listed have made a substantial, direct, and intellectual contribution to the work and approved it for publication.

## Funding

This study was supported by the National Natural Science Foundation of China (82100119, 92159302, 91859203, 81971616), the Science and Technology Project of Sichuan (2022ZDZX0018, 2020YFG0473), the Beijing Municipal Science and Technology Planning Project (Z201100005620008, Z201100005620002).

## Conflict of Interest

The authors declare that the research was conducted in the absence of any commercial or financial relationships that could be construed as a potential conflict of interest.

## Publisher’s Note

All claims expressed in this article are solely those of the authors and do not necessarily represent those of their affiliated organizations, or those of the publisher, the editors and the reviewers. Any product that may be evaluated in this article, or claim that may be made by its manufacturer, is not guaranteed or endorsed by the publisher.

## Supplementary Material

Supplementary Figure 1 The illustration of eligible patients enrolled in this study. **(A)** PD-L1 ES prediction (N=1135); **(B)** Survival cohort (N=811).

Supplementary Figure 2 Kaplan-Meier curves of overall survival prediction based on clinical model. K-M curves were stratified by **(A)** age; **(B)** sex; **(C)** smoking history; **(D)** histopathology; **(E)** PD-L1 ES; **(F)** surgery; **(G)** chemotherapy; **(H)** immunotherapy; **(I)** T-categories; **(J)** N-categories; **(K)** M-categories; **(L)** stage.
