# Supplementary figures and images for: Non-Invasive Measurement Using Deep Learning Algorithm Based on Multi-Source Features Fusion to Predict PD-L1 Expression and Survival in NSCLC
Source: Front Immunol. 2022 Apr 7;13:828560. doi: 10.3389/fimmu.2022.828560 (PMC9022118; doi:10.3389/fimmu.2022.828560)

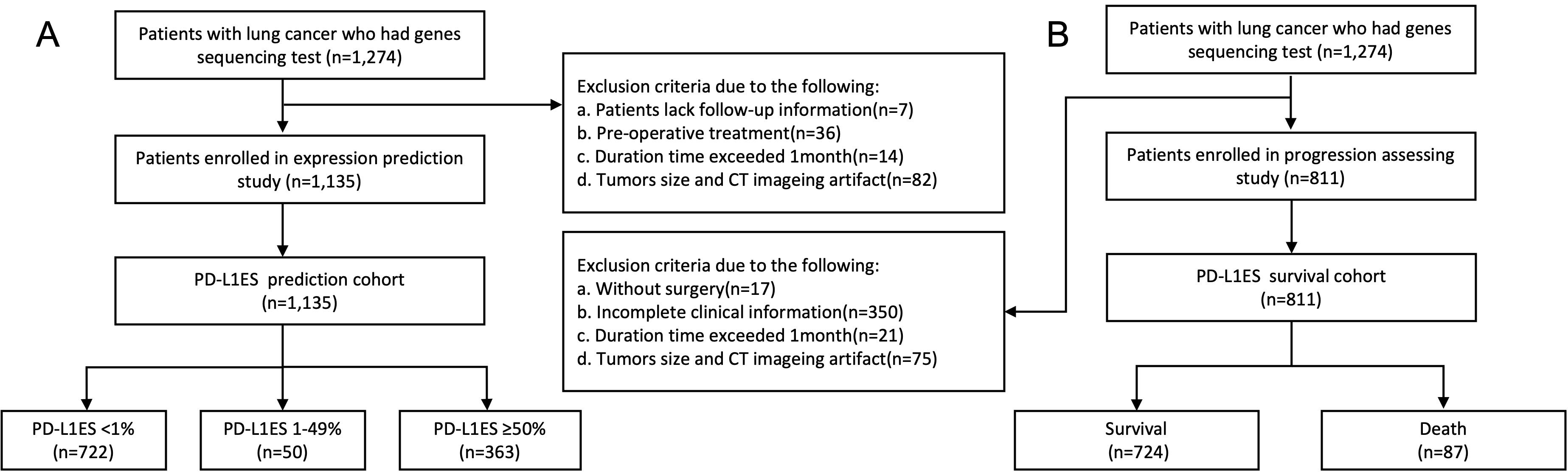

Supplement: Supplementary Figure 1 — The illustration of eligible patients enrolled in this study. (A) PD-L1 ES prediction (N=1135); (B) Survival cohort (N=811). [file Image_1.jpeg]

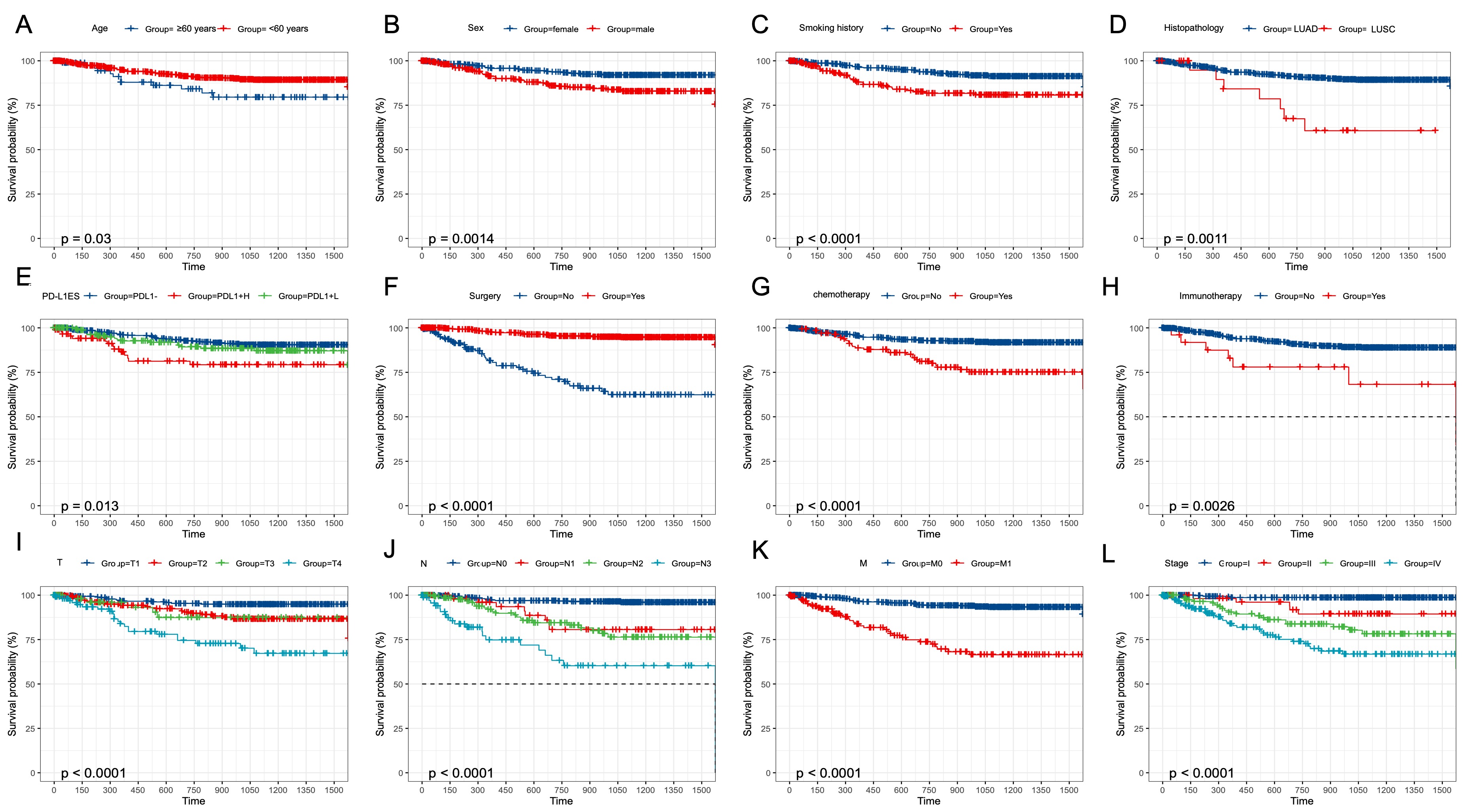

Supplement: Supplementary Figure 2 — Kaplan-Meier curves of overall survival prediction based on clinical model. K-M curves were stratified by (A) age; (B) sex; (C) smoking history; (D) histopathology; (E) PD-L1 ES; (F) surgery; (G) chemotherapy; (H) immunotherapy; (I) T-categories; (J) N-categories; (K) M-categories; (L) stage. [file Image_2.jpeg]
